# The effect of hydration state and energy balance on innate immunity of a desert reptile

**DOI:** 10.1186/1742-9994-10-23

**Published:** 2013-05-04

**Authors:** Karla T Moeller, Michael W Butler, Dale F DeNardo

**Affiliations:** 1 School of Life Sciences, Arizona State University, Tempe, AZ, USA; 2 Biology Department, Lafayette College, Easton, PA, USA

**Keywords:** Dehydration, Digestion, Energy balance, Hemagglutination, Hemolysis, Osmolality, Water

## Abstract

**Introduction:**

Immune function is a vital physiological process that is often suppressed during times of resource scarcity due to investments in other physiological systems. While energy is the typical currency that has been examined in such trade-offs, limitations of other resources may similarly lead to trade-offs that affect immune function. Specifically, water is a critical resource with profound implications for organismal ecology, yet its availability can fluctuate at local, regional, and even global levels. Despite this, the effect of osmotic state on immune function has received little attention.

**Results:**

Using agglutination and lysis assays as measures of an organism’s plasma concentration of natural antibodies and capacity for foreign cell destruction, respectively, we tested the independent effects of osmotic state, digestive state, and energy balance on innate immune function in free-ranging and laboratory populations of the Gila monster, *Heloderma suspectum*. This desert-dwelling lizard experiences dehydration and energy resource fluctuations on a seasonal basis. Dehydration was expected to decrease innate immune function, yet we found that dehydration increased lysis and agglutination abilities in both lab and field studies, a relationship that was not simply an effect of an increased concentration of immune molecules. Laboratory-based differences in digestive state were not associated with lysis or agglutination metrics, although in our field population, a loss of fat stores was correlated with an increase in lysis.

**Conclusions:**

Depending on the life history of an organism, osmotic state may have a greater influence on immune function than energy availability. Thus, consideration of osmotic state as a factor influencing immune function will likely improve our understanding of ecoimmunology and the disease dynamics of a wide range of species.

## Introduction

Immune function is vital to an animal’s survival and its subsequent fitness [1,2], yet is greatly influenced by sufficient availability of resources. Resources can become limited as a result of reduced intake or increased demand, oftentimes to simultaneously support multiple physiological functions [3]. As resource intake and demand are condition-dependent and may change temporally, immune function can vary based on environmental context [4] or season or life stage [5,6]. The importance of such factors to alterations of immune function and resulting survival has led to the recent surge of interest in ecoimmunology.

Currently, ecoimmunology research pays considerable attention to the balance of energy investment in immune defense versus other physiological functions (e.g., reproduction [3,7], sensory ability [8], exercise [9]). While energy allocation among physiological processes plays a critical role in life history trade-offs, immune function, like many other physiological functions, is not influenced solely by energetic resources. Non-energetic resources such as vitamins and trace elements can also modulate immune function [10,11].

Water, another non-energetic resource, can greatly influence organisms due to its role in biochemical or physiological processes or traits, including cellular volume and composition [12], heat shock protein production [13], plasma hormone concentrations [14], and membrane permeability [15], as well as organismal development [16]. Thus, most organisms must actively osmoregulate to maintain normal physiological function. While some taxa maintain plasma osmolality at extremes (e.g., some arthropod hemolymph: 165 mOsm/kg [17], some marine taxa: ~1000 mOsm/kg [18]), terrestrial vertebrates typically maintain osmolality between 250 and 300 mOsm [19]. However, to cope with water constraints, some species temporarily tolerate hyperosmotic states that can increase blood osmolality 20% or more (e.g., desert tortoise [20]). Seasonal dehydration occurs in various species [21,22], in a wide range of biomes, from deserts [20,23] to pelagic habitats [24], but dehydration can also occur on a daily basis (e.g., spotted salamander, *Ambystoma maculatum*[25]) or over the course of several days (e.g., camel, *Camelus dromedarius*[26]).

Changes in osmotic state affect numerous aspects of organismal function, so it is reasonable to suspect that hydration may also influence immune function. However, to our knowledge, the interaction of osmotic state with immune function has received minimal attention. Intense exercise can decrease immune response of humans [27] and periods of drought can decrease immune response of kangaroos [28], suggesting that hydric state negatively affects immune function. However, these situations likely involve simultaneous changes in energy and water balance; thus, these findings may result from a decreased investment in immune function due to either limited energy availability or physiological changes associated with osmotic stress. Due to this confound of energetic and hydration state in previous studies, the distinct effects of water balance and energy availability on immune function remain unclear.

We tested the separate effects of water balance, digestive state, and energy balance on immune function in the Gila monster, *Heloderma suspectum*, a desert-dwelling reptile that annually undergoes a period of food and water deprivation during the hot, dry season in the Sonoran Desert. As this species’ behavior seems to be more limited by hydric rather than energetic budgets [29], Christian D. Wright, unpublished observations], we hypothesized that while both hydric state and energy balance would affect immune function, hydric state would have the greater influence on immune function. Specifically, we predicted that innate immunity would be suppressed by a decrease in energetic state and by dehydration, and that this suppressed function would be more pronounced due to dehydration than to compromised energetic state. We collected plasma samples from free-ranging Gila monsters throughout the active season and laboratory-housed Gila monsters with differential access to food and water, and tested for relationships between immune function and plasma osmolality, digestive state, and fluctuation of fat stores. In particular, we assessed innate immune function using an *in vitro* assay to examine lysis and agglutination capacity of the plasma. Agglutination of foreign red blood cells is a measure of the concentration of natural antibodies (molecules that are produced prior to antigen exposure and assist in foreign particle removal and complement-mediated lysis), whereas lysis assesses the plasma’s ability to destroy foreign cells by rupturing them [30]. While this assay is not intended to capture an individual’s overall immunocompetence, it provides information regarding investment in innate immunity prior to an immune challenge and is useful when assessing innate immunity within populations [31], especially if sampling time points may be limited. Additionally, while reptiles have both innate and adaptive components of the immune system, the adaptive response is often much slower in ectotherms (by days to weeks [32]); thus, measurements of innate immunity (which acts as the first line of defense) may be the most ecologically relevant, especially if individuals have to deal with acute infection.

## Results

### Field correlations

**Figure 1:**
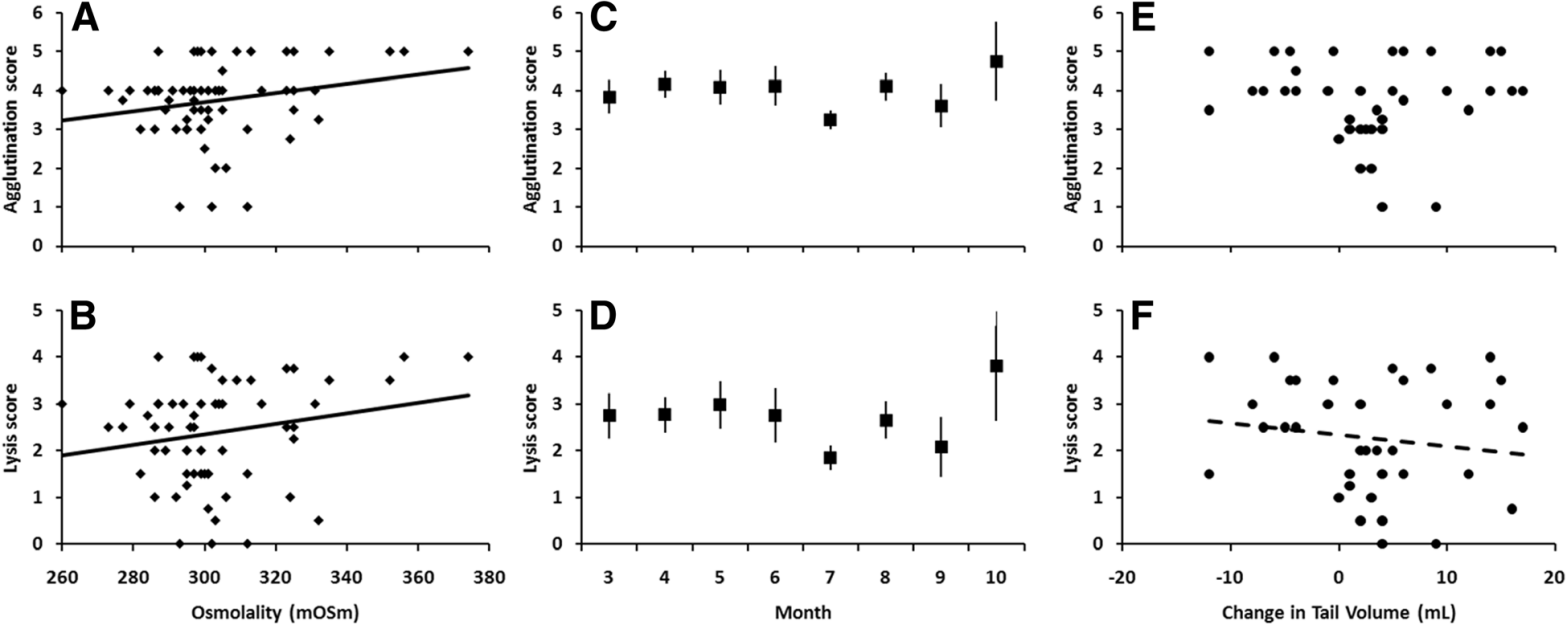
**Agglutination and lysis capacities of free-ranging Gila monsters.** Agglutination and lysis scores as a function of (**A**, **B**) plasma osmolality, (**C**, **D**) month of sampling, and (**E**, **F**) change in tail volume in free-ranging Gila monsters. Individuals with a greater plasma osmolality (i.e., more dehydrated) had greater agglutination and lysis scores. Change in tail volume (a measure of energy balance) was negatively related to lysis (F_1,21_ = 7.74, *P* = 0.011), but after removing a gravid female from analysis, this was non-significant (F_1,18_ = 3.76, *P* = 0.068), a change denoted with a dashed line. Change in tail volume had no significant relation (F_1,21_ = 3.61, *P* = 0.071) to agglutination. There was no effect of month on either agglutination or lysis ability (all *P* > 0.05). No individual was disproportionately influential (all Cook’s D < 4/n) in these analyses. Raw data are presented, uncorrected for individual. LSMeans with error bars (SE) from the mixed model are presented for month of sampling.

Month of sampling did not affect lysis (F_7,43_ = 1.55, *P* = 0.18) or agglutination (F_7,43_ = 1.66, *P* = 0.15) scores in free-ranging Gila monsters. When the single gravid female was removed from the analysis, month of sampling still did not significantly affect lysis (F_7,39_ = 1.49, *P* = 0.20) or agglutination (F_7,39_ = 1.54, *P* = 0.18). Change in tail volume was negatively related to lysis (F_1,21_ = 7.74, *P* = 0.011), yet this relationship was no longer statistically significant with the removal of the gravid female from the analysis (F_1,18_ = 3.76, *P* = 0.068). No significant relationship was detected between tail volume and agglutination (F_1,21_ = 3.61, *P* = 0.071 with gravid female; F_1,18_ = 1.32, *P* = 0.27 without gravid female). Sex did not significantly affect lysis (F_1,43_ = 0.00, *P* = 0.99) or agglutination (F_1,43_ = 0.00, *P* = 0.98) in free-ranging Gila monsters even when the gravid female was removed from analysis (lysis, F_1,39_ = 0.07, *P* = 0.79; agglutination, F_1,39_ = 0.06, *P* = 0.81). Elapsed time prior to freezing was not recorded for the field samples and thus was not included in the analysis. Despite the potential for increased variability associated with sample degradation, plasma osmolality was positively related to both lysis (F_1,43_ = 6.40, *P* = 0.015) and agglutination (F_1,43_ = 7.83, *P* = 0.0077) scores (Figure 1), and the statistical significance of the results persisted even when a gravid female was excluded (F_1,39_ = 6.17, *P* = 0.017 for lysis; F_1,39_ = 7.62, *P* = 0.0087 for agglutination). These findings support a strong association between hydric state and innate immunity even with the confound of varying (and unknown) degradation time.

### Laboratory dehydration trial

**Figure 2:**
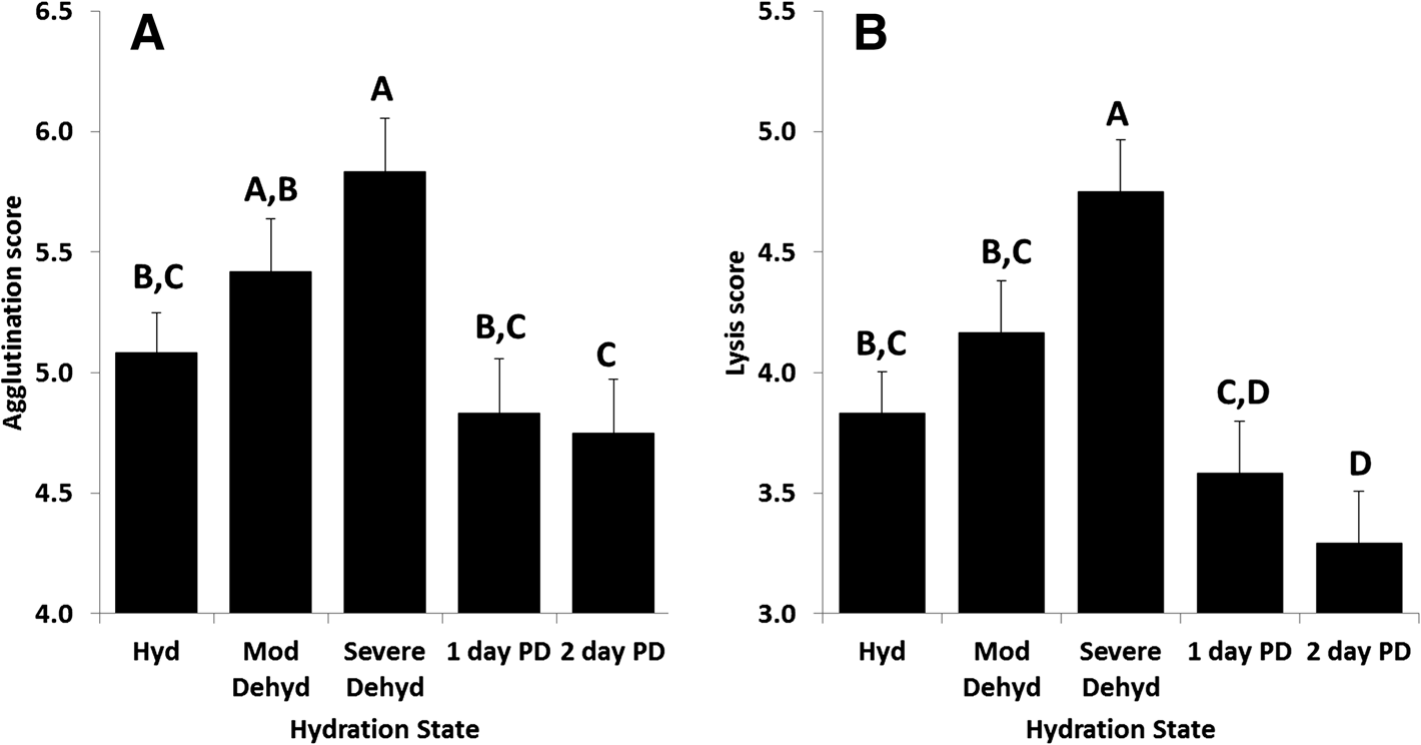
**Agglutination and lysis capacities of captive Gila monsters as a function of hydration state.** Individuals had more robust (**A**) agglutination and (**B**) lysis scores as they dehydrated, with immune function decreasing to (for agglutination) and even beyond (for lysis) baseline samples post-drinking. Here, hydration state is defined as: hydrated (270–300 mOsm); moderately dehydrated (305–335 mOsm); severely dehydrated (340+ mOsm), 1 day post-drinking (285–295 mOsm); 2 days post-drinking (255–285 mOsm). Groups that share the same letter have LSMeans that are not statistically different (*P* > 0.05). Bars represent LSMeans, with error bars showing SE.

Gila monsters took 28 ± 7 days to reach clinical dehydration, but returned to a fully hydrated state within 24 hours of drinking. Plasma osmolality over the course of dehydration and rehydration was positively related to both lysis (F_1,29_ = 34.35, *P* < 0.0001) and agglutination (F_1,29_ = 22.56, *P* < 0.0001) ability when osmolality was a continuous covariate. Sex did not affect lysis (F_1,29_ = 0.06, *P* = 0.81) or agglutination (F_1,29_ = 0.00, *P* = 0.99). Similarly, when samples were analyzed as a class variable based on dehydration state, there was a significant effect of dehydration state on both lysis (F_4,26_ = 4.65, *P* = 0.0057) and agglutination ability (F_4,26_ = 8.63, *P* = 0.0001), but no effect of sex on lysis (F_1,26_ = 0.06, *P* = 0.81) or agglutination (F_1,26_ = 0.00, *P* = 1.0). In both analyses, lytic and agglutination ability were positively related to dehydration state (Figure 2).

### Plasma dilution trial

**Figure 3:**
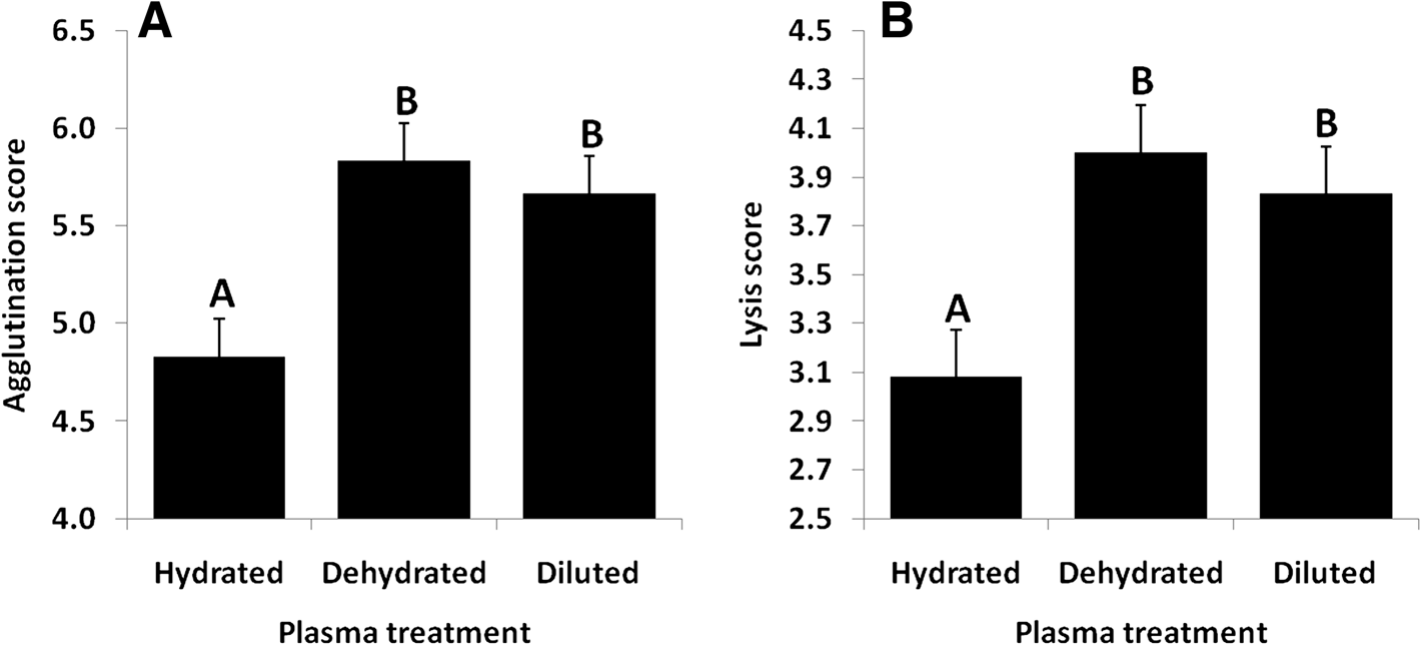
**Effects of dehydration and dilution on agglutination and lysis capacities of captive Gila monsters.** (**A**) Agglutination and (**B**) lysis scores of plasma from captive Gila monsters tested in both hydrated and dehydrated states, as well as plasma from Gila monsters in the dehydrated state that was diluted with nanopure water by 16 to 23% to match the osmolality of the individual’s hydrated plasma sample. The hydrated state samples had significantly lower agglutination and lysis scores relative to both non-manipulated and diluted dehydrated samples. Groups that share the same letter have LSMeans that are not statistically different (*P* > 0.05). Bars represent LSMeans, with error bars showing SE.

Plasma treatment (hydrated animal, dehydrated animal, diluted plasma from dehydrated animal) significantly affected both lysis (F_2,4_ = 12.12, *P* = 0.020) and agglutination (F_2,4_ = 7.75, *P* = 0.042) scores. For both metrics of immune function, hydrated samples had statistically lower performance than both dehydrated and diluted samples (Figure 3). Lysis data were normally distributed (*P* = 0.57), but agglutination data were not (*P* = 0.026), thus we suggest applying this result with caution. Effects of sex were not tested because we used only one female and three males, which would lead to erroneous statistical conclusions.

### Digestive state trial

**Figure 4:**
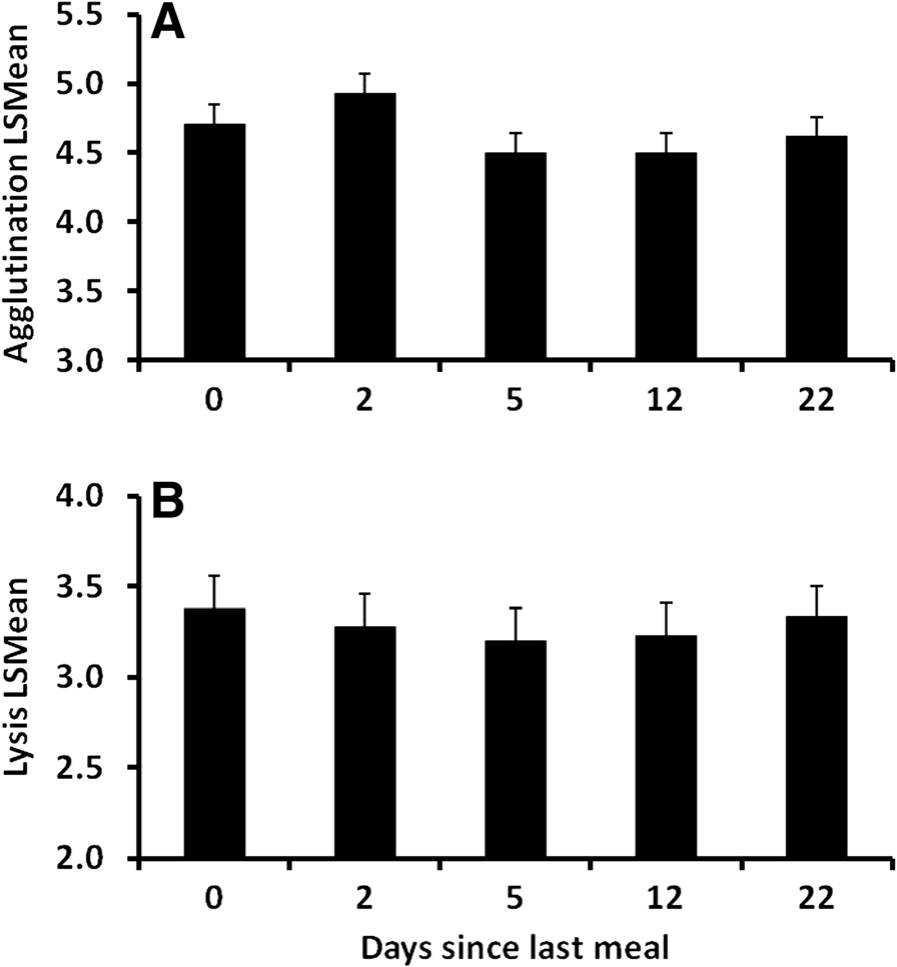
**Effects of digestive state on agglutination and lysis capacities.** (**A**) Agglutination and (**B**) lysis scores from captive Gila monsters as a function of digestive state. Individuals were given a meal (approximately 20% of body mass) immediately after blood collection on day 0. Neither agglutination nor lysis scores significantly varied (*P* < 0.05) over the duration of meal absorption. Bars show LSMeans and error bars represent SE.

Neither lysis (F_4,34_ = 0.30, *P* = 0.88) nor agglutination (F_4,34_ = 0.94, *P* = 0.45) ability changed with digestive state (Figure 4). Sex did not affect analysis for either lysis (F_1,8_ = 1.39, *P* = 0.27) or agglutination (F_1,8_ = 2.95, *P* = 0.12).

### Sample degradation

**Figure 5:**
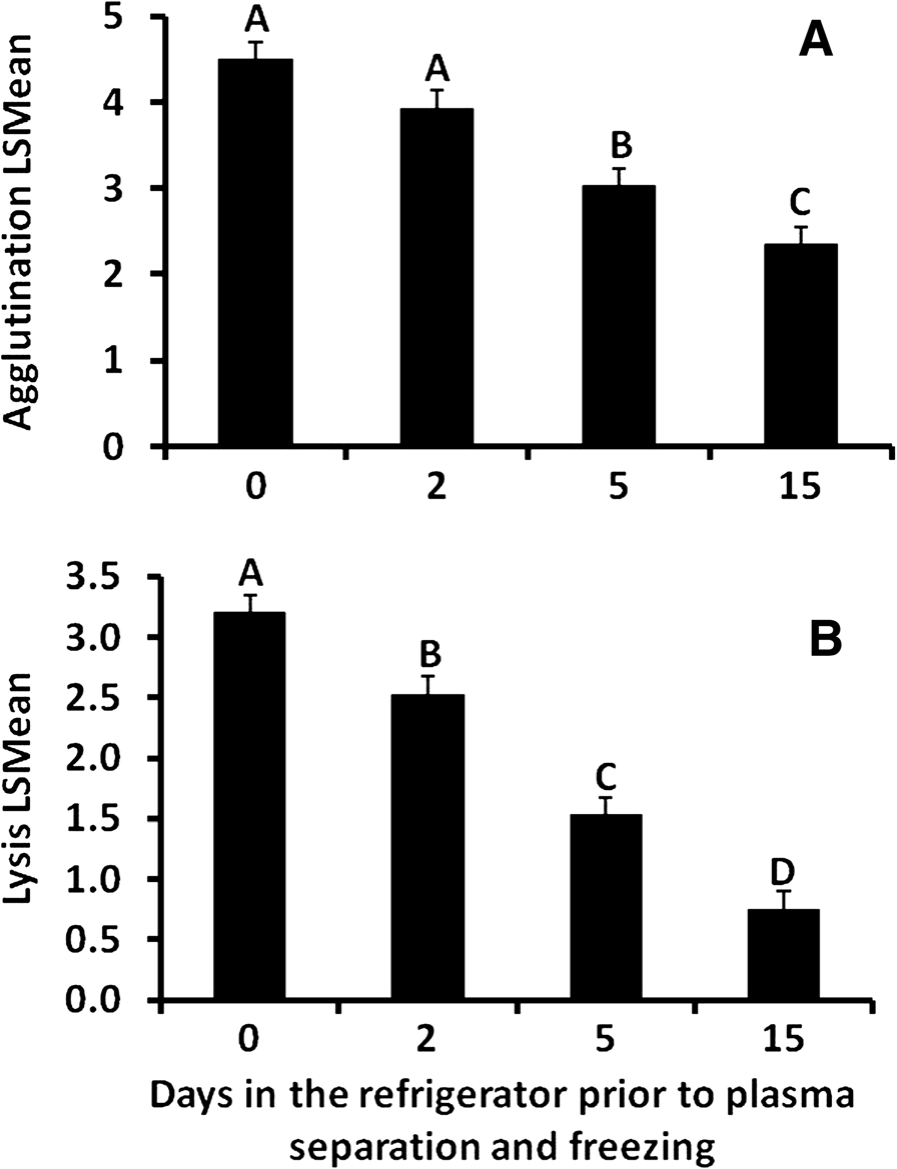
**Degradation of agglutination and lysis capacities over time.** Number of days in the refrigerator prior to plasma separation and freezing significantly reduced both (**A**) agglutination and (**B**) lysis scores. Groups that share the same letter have LSMeans that are not statistically different (all *P* < 0.05), although agglutination scores on days 0 and 2 approached significance (*P* = 0.058). Bars show LSMeans and error bars represent SE.

Samples degraded over time when left in the refrigerator prior to freezing, with both lysis (F_3,24_ = 44.52, *P* < 0.0001) and agglutination (F_3,24_ = 19.10, *P* < 0.0001) scores decreasing (Figure 5; see Additional file 1 for further discussion). Post-hoc tests of LSMeans revealed that agglutination values were significantly lower after 5 and 15 days of refrigeration (both *P* < 0.0001), and lysis scores were significantly lower after 2, 5, and 15 days of refrigeration (all *P* < 0.013). Sex affected lysis (F_1,8_ = 11.05, *P* = 0.0105) and agglutination (F_1,8_ = 12.42, *P* = 0.0078), but there was no significant interaction between sex and day for either lysis (F_3,24_ = 0.84, *P* = 0.49) or agglutination (F_3,24_ = 3.74, *P* = 0.025). Agglutination of samples from females was greater than that of males on days 2 (*P* = 0.0472) and 15 (*P* < 0.0001; LSMeans F: 3.75, SE: 0.329; M 1.75, SE: 0.215). Due to the low number of females in our analysis (3 females to 7 males), we did not interpret these sex differences, but encourage including sex as a factor in future studies of sample degradation.

## Discussion

Previous studies have examined the effect of dehydration on some physiological systems [12,16], but exploration of the effects of dehydration on the immune system has been relatively rare. Our data demonstrate that osmotic state can alter immune function, and does so in a surprising fashion; despite our expectations of a decreased immune response during times of dehydration, we consistently observed that Gila monsters have more robust innate immune function during bouts of dehydration. Specifically, our results from both field and laboratory experiments show a strong, positive correlation between plasma osmolality and lysis and agglutination capacity of plasma on both continuous and discrete scales of hydration state (Figures 1 and 2, respectively). Though isolated assessments of immune function do not fully represent the suite of alterations in immunity that may be occurring within an organism, as different combinations of protective measures can achieve the same level of defense [31], the increased response of plasma during dehydration indicates that the innate humoral immune system exhibits increased reactivity during these times. These findings provide a new context that will likely enhance the general understanding of the influences of resource limitations on the physiology and ecology of a wide range of species.

A corresponding increase in immune response and osmolality may serve to prime the immune system with a more robust first line of defense during times of the greatest osmotic stress, when mounting a more expensive response may be impractical. In organisms that do not tightly regulate plasma volume, such a response could be solely a function of the concentration of immune molecules (e.g., natural antibodies) within the plasma. In dehydration trials, osmolality increased with a concurrent decrease in body mass, indicative of decreasing plasma volume (and potentially a concentrating effect). However, diluting dehydrated samples back to the osmolality of hydrated samples did not decrease the plasma capacity for agglutination and lysis to the levels expected if concentration were the sole influencing factor. This suggests that increased immune function is not strictly bound by the increases in solute concentrations associated with dehydration, but is rather augmented by an additional physiological effect that may be employed during times of osmotic stress.

While this study did not explicitly identify the mechanistic reasons behind the relationship between osmolality and immune function, it did lead to several functional hypotheses. The increase in agglutination and lysis ability could be due to an increased investment in less energetically expensive, innate immune defenses [1] during times of osmotic stress. While the data regarding the role of osmolality and osmotic stress in immune cell function are currently lacking, it is reasonable that immune cells are negatively affected by dehydration just like other cell types [33]. Thus, an investment in proteins that can perform basic immune functions during periods of osmotic stress while reducing investment in cellular processes (e.g., phagocytosis, cell-mediated immune responses) could be evolutionarily adaptive. Indeed, shifts in utilization of different components of the immune system based on the environment have been documented previously in fish, with innate immune function being utilized more readily at lower temperatures, whereas acquired immunity is relied upon at higher temperatures [34]. The correlation of lysis and agglutination scores within a sample (r = 0.93, *P* < 0.0001) may support the use of basic immune functions during osmotic stress. While this correlation is not uncommon, these measures are not always positively correlated [35]; additional immune assessments may help clarify the importance of this finding.

The purpose of the increased immune function we observed also remains to be experimentally investigated; for example, it is possible that these higher metrics provide increased protection against pathogens that are more prevalent during the dry season, or that the individual is reallocating resources to a less effective, but physiologically cheaper, method of immune defense during a stressful period. While immune function and water balance may be physiologically linked (e.g., non-specific protein kinases are involved in signaling pathways for both immune function and cell volume regulation [36]; keratinocytes contribute to both immune defense and water conservation [37,38]), we still lack adequate understanding of how the interaction of immune function and hydration affects organismal ecology.

Osmolality-based differences in immune function may have profound ecological effects, through, for example, alteration of disease transmission. In some species, communal conditions may occur during times of osmotic stress, creating ample opportunity for disease transmission, such as in animals that use limited subterranean refugia or those that live in areas where preferred habitats become increasingly patchy during the dry season [39,40]. Many desert tortoise populations in the Mojave Desert have declined due to increased mortality rates resulting from upper respiratory tract infections [41]. Additionally, the disease-causing fungus *Batrachochytrium dendrobatidis* associated with amphibian declines appears to more strongly decrease population size during dry seasons that have an increased number of dry spells [40]. Estimates of the force of infection (i.e., probability of transmission) may be altered if the hot, dry season (during which animals experience osmotic stress) affects immune function, either severely increasing or decreasing rates of transmission, thereby making individuals more susceptible to infection on a seasonal cycle.

For species that experience regular or prolonged periods of dehydration, any effects of increased osmolality on immune function could affect individual survival. Gila monsters have high rates of injury in some populations (at a natural and an urban site, scarring was observed in roughly 32% and 71% of adult individuals, respectively; Jon R. Davis, unpublished observations), likely making immunity an important investment even during periods of dehydration. Additionally, in the wild, dehydration bouts are frequently nested within season, and seasonal investment in immune function may be adaptive if there is a predictable seasonal variation in pathogen prevalence or virulence. However, the lack of a monthly effect in our field study, as well as the rapid reduction in innate immune function after rehydration in the lab demonstrates that the variation in innate immunity associated with hydration state is not a result of seasonal adjustments in immune strategies.

While our results are directly relevant to animals that dehydrate seasonally (including periods of drought and other resource-limited seasons, such as winter, when many animals are dormant), these results may also be relevant to animals that experience changes in hydration state on shorter time scales, as was observed during rehydration trials. Thus, fluctuations in osmotic state may be an important factor to include in estimates of disease transmission or general efforts to understand the ecoimmunology of various species. Further investigation regarding how short-term variation in hydration state may affect immune function in a more ecologically relevant context (e.g., during wound healing) will increase our understanding of the implications of these results.

In contrast to our finding that immune function varied with hydration state, we found no evidence that digestive state affected immune function. Immune responses are energetically expensive [42] and can result in an increased metabolic load [43]. However, an immune response is frequently accompanied by the induction of anorexia, which may allow for the utilization of physiological pathways that enhance immune function [44], highlighting the context-dependent relationship between energy acquisition and immune function. While food intake has been shown to affect the immune response in other lizards during times of high energy demand [7], Gila monsters may be less sensitive to food intake because their life history includes frequent extended bouts of aphagia and thus they rely heavily on energy stores to support physiological functions. Such species may be more likely to maintain a constant investment in immune function as long as adequate energy stores exist.

Changes in tail volume provide us with a metric for relative energy stores in individual Gila monsters. An increase in tail volume, indicative of increased energy stores and positive energy balance, was negatively associated with lysis, but not agglutination. While the exclusion of a gravid female made this finding non-significant (*P* = 0.068), the suggested trend of decreasing lysis with increasing tail volume may indicate a trade-off between innate and cell-mediated immune responses, as potential differences in costs and benefits may alter the advantage of these respective responses with ecological context [1,34]. Further research to test this idea is needed and must be reconciled with the lack of effect on agglutination and the potential influence of reproductive states. The lack of correlation between lysis or agglutination and change in tail volume, in conjunction with the finding that changes in energetic state over shorter time periods (i.e., in the lab feeding trial) did not affect agglutination or lysis, suggests that ecologically relevant variation in energetic intake or balance has a minimal effect on these measures of innate immunity, at least in this species. Because we detected differences in immune function due to osmotic state, future studies evaluating various immune metrics during periods of positive and negative energy balance as well as during different hydration states will help clarify how apparently complex immune strategies [31] are associated with both energy and water availability.

## Conclusions

According to the present understanding of ecoimmunology, availability of energetic resources is a primary factor influencing the immune response of individuals across numerous taxa. However, using both laboratory and field studies, we show that osmotic state can have a greater influence on immune function than digestive state or fluctuations in the extent of energy stores. It remains unclear whether dehydration-induced shifts occur differently between specific components of the immune system or whether the entire immune system is systemically suppressed or enhanced, and studies to test this possibility are greatly needed. Overall, this study emphasizes the need for further research into the effects of hydric state on immune function; this relationship may have important implications for understanding the causes of individual variation in ecoimmunology, as well as changes in rates of disease transmission in some species.

## Materials and methods

### Study animals

The Gila monster is a large-bodied, desert-dwelling lizard that has storage capacities that provide water reserves (in the urinary bladder) that can last up to 3 months and energy reserves (in coelomic and tail fat bodies) that last even longer [45,46]. These reserves enable Gila monsters to balance resource acquisition and expenditure over abnormally long periods relative to other lizards. Despite these extensive storage capacities, Gila monsters experience seasonal resource imbalances that can alter behavior patterns. For example, dehydration causes Gila monsters to reduce surface activity during the hot, dry season [29].

### Sample collection

Blood samples were drawn from the caudal vein using heparinized 1 ml syringes. Plasma was separated from cells via centrifugation at 3000 rpm for 3 minutes and frozen at −80°C within 1 hour, with the exception of samples for the degradation trials (see below) and field samples. Field samples were collected at the beginning of each month of the active season (April through September) from 17 radio-tagged, free-ranging Gila monsters (9 females and 8 males). One female was gravid, so separate analyses were run including and excluding this individual. Due to reduced activity during certain periods, not all animals were sampled every month. Blood samples were collected within 5 minutes of capture and whole blood was placed on ice within 1 hour of sampling. As some trips to the field were longer than others, samples remained on ice for anywhere from 3 hours to 2 days before plasma was separated and frozen at −80°C.

### Field-based correlation between hydration state and immune function

In 2010, we collected plasma samples from 17 free-ranging Gila monsters from a population in the Arizona Upland subdivision of the Sonoran Desert, roughly 30 km NNE of Tucson. Adult Gila monsters (mean body mass = 396 g; range = 243 – 552 g) were implanted with 13 g radiotransmitters (model SI-2, Holohil Systems Ltd., Ontario, Canada; see [29] for procedure description) and tracked at least weekly. To evaluate energy balance, variation in tail volume (±1 ml) was recorded using water displacement whenever a plasma sample was acquired from an animal. Serial tail volume measurements provide an effective index of changes in an individual’s stored energy over time because Gila monsters store energy reserves caudally [45,47] and tail volume is not influenced by fecal elimination or water intake [46]. We determined plasma osmolality and performed agglutination and lysis assays on aliquots of each field sample.

### Laboratory trials

All other studies were conducted in the laboratory in 2010 and 2011 using a long-term captive population of wild-caught Gila monsters (mean mass = 497 g; range = 420 – 602 g). These Gila monsters were typically housed in individual cages (35 × 75 × 12.5 cm; Freedom Breeder, Turlock, California, USA) where they had continuous access to water, refugia, and a thermal gradient (25 to 35°C). For dilution trials (see below), animals were housed as described, but with no access to water. During the initial dehydration trial (see below), animals were housed in individual cages (24 × 36 × 13 cm; United States Plastic Corporation, Lima, Ohio, USA) with a modified screen top within an environmental chamber held at a constant 30°C, which approximates the species’ preferred body temperature [45]. During this trial, the animals had no access to water or refugia. All laboratory Gila monsters were maintained in good body condition on a diet of adult mice, but were fasted for at least 14 days before the beginning of each trial to attain a post-absorptive state and thus standardize digestive state. All females were non-reproductive, as confirmed using ultrasound. Digestive and degradation trials involved 10 animals (each trial used the same three females and seven males), and the laboratory dehydration trials involved a subset of six of these animals (one female and five males). Four Gila monsters were used in the dilution trial (one female and three males), two of which were also used in all other laboratory trials. Gila monsters used in multiple trials had at least 2 weeks of water access between trials.

### Laboratory dehydration trial

For initial dehydration trials, six animals had their bladders drained via transurethral catheterization as described in Davis and DeNardo [46]. They were then housed within an environmental chamber (30°C air temperature, affluent air at 2°C dew point) in individual containers. To monitor plasma osmolality, each animal was bled (0.15 ml) within 2 hours of catheterization, once more during the first week (0.07 ml), and twice per week (0.07 ml) thereafter until the animal reached a state of moderate dehydration (plasma osmolality of 305–335 mOsm). At this point, a second 0.15 ml sample was obtained. Plasma continued to be monitored twice per week until the animal showed clinical signs of severe dehydration (e.g., lethargy, plasma osmolality above 340 mOsm). Once severely dehydrated, a third 0.15 ml blood sample was taken, and the animal was provided with water to rehydrate. Two final 0.15 ml blood samples were taken 24 and 48 hours post-rehydration. We used the initial, moderately dehydrated, severely dehydrated, 24-hour post rehydration, and 48-hour post rehydration samples in agglutination and lysis assays to evaluate the effect of hydration state on innate immunity.

### Plasma dilution trial

Dilution trials were conducted to test whether any differences in immune function due to hydration state of the animal could be explained purely by plasma osmolality during assaying. An initial 0.15 ml blood sample was collected from four laboratory animals for osmolality and lysis and agglutination analyses. Animals were held without food or water, and body masses were closely monitored as a rough indicator of water loss (since energy loss over this time would be minimal). Additionally, blood samples (0.07 ml) were taken twice per week to monitor plasma osmolality. Once an animal had lost 15% of its initial body mass or had reached a plasma osmolality of over 320 mOsm (reaching moderate to severe dehydration according to clinical signs shown by some Gila monsters in this hydration state), a final 0.15 ml blood sample was collected from the animal. An aliquot of plasma (60 μl, with the exception of one 30 μl aliquot from a sample that required a repeated osmolality analysis) from each of these dehydrated state samples was then diluted with nanopure water (5 to 15 μl) to generate a sample from a dehydrated animal but with an osmolality similar to that of the animal’s plasma when in a hydrated state. We used the initial hydrated (20 μl plasma), the dehydrated (20 μl plasma), and the diluted dehydrated (15.4-16.8 μl plasma with 3.2-4.6 μl nanopure to make 20 μl total) samples from each individual to compare osmolality, lysis, and agglutination.

### Digestive state trial

Effects of digestive state were assessed using 10 adult Gila monsters. After an initial blood sample (0.07 ml, day 0) was collected, each animal was fed a mouse meal equal to 20.0 ± 0.5% of its body mass. Additional 0.07 ml blood samples were then collected at day 2 (representing peak metabolic investment into digestion), day 5 (non-peak investment into digestion), day 12 (post-absorptive with minimal to no investment into digestion), and day 20 (when animals were definitively post-absorptive), based on Christel, DeNardo, and Secor [48].

### Sample degradation trial

As the blood samples for the field component of this study were initially collected for a separate study for which immune molecule degradation was not a concern, time elapsed prior to freezing of samples was not recorded. To test for possible effects of sample storage time on the immune assays, we collected blood samples from 10 Gila monsters and refrigerated the samples immediately at 2°C to mimic conditions that some field samples experienced. Whole blood aliquots were removed from the refrigerated sample 0 (immediately), 2, 5, and 15 days later, plasma was separated from aliquots, and plasma was frozen at −80°C until analyzed. These sample points represent a range of degradation times, including those our laboratory samples were subjected to (immediately: 0 days), those which our field samples were subjected to (0 to 2 days), as well as time points (5 and 15 days) beyond the degradation time experienced by any of our samples.

### Sample processing: determination of plasma osmolality

Plasma osmolality was determined for all samples except those from the digestive state and sample degradation trials. During the latter trials, water was provided throughout the trial in order to assure a normosmotic state and thus enable us to tease apart the effects of digestive state or degradation from those due to dehydration. Osmolalities were determined in triplicate using a vapor pressure osmometer (±6 mOsm/kg; model 5500×r; Wescor, Inc., Logan, Utah, USA) as described in Davis and DeNardo [46].

### Sample processing: natural antibody agglutination and lysis assay

To assess natural antibody agglutination and lysis of all samples from all trials, we followed the protocol of Matson et al. [30] with several modifications. Briefly, 20 μl of each plasma sample were serially diluted from 1:2 to 1:2048 with phosphate-buffered saline (PBS) along a row of a 96-well plate. Plasma was not added to the PBS in the final row in the well to act as a negative control. We then added 20 μl of 50% heparinized whole sheep blood (SBH050, HemoStat Laboratories, Dixon, California, USA) diluted 1:100 to each well. Each plate was gently vortexed, covered with Parafilm, and because temperature can significantly affect lysis and agglutination scores [49], we incubated each plate for 90 minutes in a 29°C water bath to approximate the mean diurnal temperature of free-ranging Gila monsters during the active season [45]. To improve visualization, we then tilted the plates at 45° for 20 minutes at room temperature [30], after which they were scanned at 600 dots per inch using a flat-bed scanner (Hewlett-Packard Co., ScanJet 3670) for agglutination images. The plates were then left flat at room temperature for 70 minutes, after which they were rescanned for lysis images (see [30] for scoring procedures). Scanned images of the plates were visually scored by KTM and MWB independently. Each scorer selected the well along each row in which definitive agglutination (denoted by preventing the formation of a dense RBC pellet) or lysis (denoted by the lack of compartmentalization of red pigment) had occurred; both KTM and MWB’s scores were repeatable (see Lessells and Boag [50] for repeatability analysis procedure) for both agglutination (R = 0.99, *P* < 0.0001) and lysis (R = 0.96, *P* < 0.0001). Thus, we used average values for all subsequent analyses. To determine repeatability of samples among plates, we also ran aliquots of six samples on each of five different plates, and these samples were significantly repeatable for both agglutination (R = 0.83, *P* < 0.0001) and lysis (R = 0.88, *P* < 0.0001).

### Statistical analyses

To test for the effects of season, body condition, and osmolality on lysis and agglutination in animals from the field, we performed a mixed model analysis with either osmolality or change in tail volume as a covariate, month of capture as a fixed effect, and individual as a random effect. Since animals from the laboratory dehydration trials dehydrated at different rates and were therefore at different hydration states on different days, we tested for effects of hydric state on either lysis or agglutination using two different mixed models, with either the continuous variable of osmolality or the class variable of hydration state (hydrated, 270–300 mOsm; moderately dehydrated, 305–335 mOsm; dehydrated, 340+ mOsm, rehydrated, 1 day post-drinking; rehydrated, 2 days post-drinking) as the independent variable, individual as a random effect, and either lysis or agglutination score as the dependent variable. To directly test the effects of plasma osmolality on lysis and, separately, agglutination, we performed repeated measures analyses of variance (rmANOVAs) on scores from hydrated, dehydrated, and diluted plasma samples from each of four individuals. We also used rmANOVAs to test for the effects of digestive state and degradation on either lysis or agglutination scores. All statistics were performed using SAS 9.2 (Cary, NC) and least squares means (LSMeans) are reported ± standard error.

## Competing interests

The authors declare that they have no competing interests.

## Authors’ contributions

KTM conceived of the study and drafted the manuscript, KTM, MWB, and DFD designed the study and revised the manuscript, KTM collected samples, KTM and MWB performed laboratory analyses, and MWB performed statistical analyses. All authors approved the final manuscript.

## Supplementary Material

Additional file 1 Details regarding sample degradation. Click here for file
