# Supplementary material for: The effect of hydration state and energy balance on innate immunity of a desert reptile
Source: Front Zool. 2013 May 4;10:23. doi: 10.1186/1742-9994-10-23 (PMC3660207; doi:10.1186/1742-9994-10-23)
Supplement: Additional file 1 — Details regarding sample degradation. [file 1742-9994-10-23-S1.docx]

**Supporting Information 1:** *Details regarding sample degradation*

We found that agglutination and lysis abilities of plasma degraded when the samples were kept on ice and not quickly frozen. It is unlikely that these differences were due to changes in osmolality of the plasma, as the samples were stored in air-tight containers, yielding minimal opportunity for evaporation to occur, and our dilution results indicate agglutination and lytic capabilities were not altered by concentration effects. Therefore, it is likely that the natural antibodies and/or complement proteins themselves were degrading when not frozen. As this difference manifested within just 48 hours, we recommend the centrifugation and freezing of plasma on the same day, if not within a few hours, of collection. However, the specific rate of sample degradation on a finer scale (hours) has yet to be examined, so we cannot provide a more specific recommendation.
